# Metabolic Analyses Revealed Time-Dependent Synergistic Killing by Colistin and Aztreonam Combination Against Multidrug-Resistant *Acinetobacter baumannii*

**DOI:** 10.3389/fmicb.2018.02776

**Published:** 2018-11-16

**Authors:** Mei-Ling Han, Xiaofen Liu, Tony Velkov, Yu-Wei Lin, Yan Zhu, Mengyao Li, Heidi H. Yu, Zhihui Zhou, Darren J. Creek, Jing Zhang, Jian Li

**Affiliations:** ^1^Biomedicine Discovery Institute, Infection and Immunity Program, Department of Microbiology, Monash University, Clayton, VIC, Australia; ^2^Institute of Antibiotics, Huashan Hospital, Fudan University, Shanghai, China; ^3^Department of Pharmacology & Therapeutics, School of Biomedical Sciences, Faculty of Medicine, Dentistry and Health Sciences, The University of Melbourne, Parkville, VIC, Australia; ^4^Department of Infectious Diseases, Sir Run Run Shaw Hospital, Zhejiang University School of Medicine, Hangzhou, China; ^5^Drug Delivery, Disposition and Dynamics, Monash Institute of Pharmaceutical Sciences, Monash University, Parkville, VIC, Australia

**Keywords:** polymyxin, beta-lactam, combination therapy, lipopolysaccharide, peptidoglycan, metabolomics

## Abstract

**Background:** Polymyxins are a last-line class of antibiotics against multidrug-resistant *Acinetobacter baumannii*; however, polymyxin resistance can emerge with monotherapy. Therefore, synergistic combination therapy is a crucial strategy to reduce polymyxin resistance.

**Methods:** This study conducted untargeted metabolomics to investigate metabolic responses of a multidrug-resistant (MDR) *A. baumannii* clinical isolate, AB090342, to colistin and aztreonam alone, and their combination at 1, 4, and 24 h. Metabolomics data were analyzed using univariate and multivariate statistics; metabolites showing ≥ 2-fold changes were subjected to bioinformatics analysis.

**Results:** The synergistic action of colistin-aztreonam combination was initially driven by colistin via significant disruption of bacterial cell envelope, with decreased phospholipid and fatty acid levels at 1 h. Cell wall biosynthesis was inhibited at 4 and 24 h by aztreonam alone and the combination as shown by the decreased levels of two amino sugars, UDP-*N*-acetylglucosamine and UDP-*N*-acetylmuramate; these results suggested that aztreonam was primarily responsible for the synergistic killing at later time points. Moreover, aztreonam alone and the combination significantly depleted pentose phosphate pathway, amino acid, peptide and nucleotide metabolism, but elevated fatty acid and key phospholipid levels. Collectively, the combination synergy between colistin and aztreonam was mainly due to the inhibition of cell envelope biosynthesis via different metabolic perturbations.

**Conclusion:** This metabolomics study is the first to elucidate multiple cellular pathways associated with the time-dependent synergistic action of colistin-aztreonam combination against MDR *A. baumannii*. Our results provide important mechanistic insights into optimizing synergistic colistin combinations in patients.

## Introduction

Multidrug-resistant (MDR) *Acinetobacter baumannii* is an important nosocomial pathogen and can cause ventilator-related pneumonia, bloodstream infections, urinary tract infections and meningitis (Dijkshoorn et al., 2007; Fishbain and Peleg, 2010). It has become very problematic due to rapid development of resistance to all currently available antibiotics, including *β*-lactams (Perez et al., 2007; Mak et al., 2008; Boucher et al., 2009). Without novel classes of antibiotics in the near future, polymyxins (i.e., polymyxin B and colistin) have resurged as a last-resort therapy against MDR *A. baumannii* (Zavascki et al., 2007; Lim et al., 2010; Sampson et al., 2012). Polymyxins kill bacterial cells via an initial electrostatic interaction between the positively charged L-*α*,*γ*-diaminobutyric acid (Dab) residues of polymyxins and the negatively charged phosphate groups of lipopolysaccharide (LPS) in Gram-negative outer membrane (OM) (Velkov et al., 2010). This is followed by non-polar interactions which allow the hydrophobic moieties (*N*-terminal fatty acyl tail and D-Phe^6^-L-Leu^7^) of polymyxins to penetrate into the OM, disorganize the cell envelope, and result in cell death (Velkov et al., 2010, 2013; Yu et al., 2015; Rabanal and Cajal, 2017). However, the exact mechanism of polymyxin killing is still not clear.

Unfortunately, *A. baumannii* can develop resistance to polymyxins through covalent modifications of lipid A phosphate groups with positively charged moieties [e.g., phosphoethanolamine (pEtN) and galatosamine (GalN)] or by the complete loss of LPS (Moffatt et al., 2010; Arroyo et al., 2011; Henry et al., 2011; Boll et al., 2015). These modifications significantly reduce the net negative charge on the bacterial membrane and repel the binding to polymyxins. Therefore, to reduce the emergence of polymyxin resistance, combination therapies of polymyxins with other antibiotics are strongly recommended (Karageorgopoulos and Falagas, 2008; Cai et al., 2012; Henry et al., 2015; Nation et al., 2015; Maifiah et al., 2017). A number of *in vitro* and *in vivo* studies and clinical case reports have proposed synergistic colistin combination therapies against heteroresistant *A. baumannii* isolates to prevent the development of colistin resistance (Montero et al., 2002; Yoon et al., 2004; Vidaillac et al., 2012; Bae et al., 2016). Aztreonam was approved by FDA in 1986 and is the only clinically available monobactam against aerobic Gram-negative bacteria. In the context of the global spread of MDR Gram-negatives, the pharmacokinetics and pharmacodynamics of aztreonam have been re-investigated recently (Eliopoulos and Bush, 2001; Ramsey and MacGowan, 2016). However, aztreonam monotherapy can be problematic due to the degradation by *β*-lactamases including extended-spectrum *β*-lactamases (ESBLs), AmpC type *β*-lactamase and *Klebsiella pneumonia* carbapenemases (KPCs) in Gram-negative bacteria, which has promoted interest in combination therapies (Gutmann et al., 1988; Ramsey and MacGowan, 2016). In a recent study, using a multiple-combination bactericidal test, the combination of colistin and aztreonam showed synergistic effect against a number of colistin-resistant *A. baumannii* clinical strains (Bae et al., 2016). However, the mechanism that underlies the synergistic killing of the colistin-aztreonam combination has not been fully investigated.

Systems pharmacology has been extensively used for understanding bacterial physiology and mechanisms of antibiotic killing and resistance (Henry et al., 2015; Maifiah et al., 2017; Zampieri et al., 2017; Zhu et al., 2018). In particular, metabolomics provides a powerful systems tool to identify and quantify key intracellular metabolites at the network level in responses to antibiotics (Kaddurah-Daouk et al., 2008; Maifiah et al., 2017; Zampieri et al., 2017). In the present study, comparative metabolomics was conducted to elucidate the mechanism of the synergistic colistin-aztreonam combination against *A. baumannii*. Our findings provide important insights into the optimization of this important combination for the treatment of MDR *A. baumannii* infections.

## Materials and Methods

### Strain, Antibiotics and Reagents

*Acinetobacter baumannii* AB090342 was collected in a clinical study approved by the institutional review board of Sir Run Run Shaw Hospital, Zhejiang, China. Written informed consent was obtained from a 60-year-old male patient who was given intravenous colistimethate sodium (150 mg colistin base activity every 12 h for 10 days) for the treatment of ventilator-associated pneumonia. The patient showed clinical and microbiological failure and *A. baumannii* AB090342 was isolated from the sputum. The isolate was identified using 16S ribosomal DNA sequencing and multilocus sequence typing (MLST). *A. baumannii* AB090342 was colistin susceptible (MIC = 0.5 mg/L), while aztreonam resistant (MIC > 128 mg/L.)

Colistin (sulfate, CAS# 1264-72-8) and aztreonam (CAS# 78110-38-0) were purchased from Sigma-Aldrich (Saint Louis, United States). Antibiotic solutions were prepared before the metabolomics study using Milli-Q water (Millipore Australia, North Ryde, NSW, Australia) and filtered through 0.22-μm syringe filters (Sartorius, Melbourne, VIC, Australia).

### Genome Sequencing

The genomic DNA was extracted using a Genomic DNA Purification Kit (Tiangen, Beijing, China) according to the producer instruction and stored at -80°C before sequencing. A 300-bp paired-end library was constructed with the purified DNA sample following the standard Illumina paired-end protocol. Cluster generation was performed in C-bot and sequencing was performed on Illumina Hiseq2500 (Illumina, San Diego, CL, United States) with 150 cycles. Draft genome was assembled using Velvet (Ver 1.0.15) (Delcher et al., 2007; Zerbino and Birney, 2008). Raw data of AB090342 was aligned to the AB307-0294 genome (Genbank Accession: NC_011595) and single nucleotide polymorphisms (SNPs) determined by Velvet (Langmead and Salzberg, 2012).

### Bacterial Culture for Metabolomic Experiments

Prior to experiments, *A. baumannii* AB090342 was subcultured on nutrient agar plates and incubated for 16–18 h at 37°C. A single colony was then inoculated into 10 mL of cation-adjusted Mueller-Hinton broth (CaMHB, Oxoid) and incubated in a shaking water bath at 180 rpm and 37°C for 18 h. The overnight culture was then diluted by 1:100 into four different reservoirs with 100 mL fresh CaMHB and grown to an optical density at 600 nm (OD_600_) of 0.50 ± 0.02 to achieve the starting inoculum at ∼10^8^ cfu/mL at early logarithmic growth phase. The bacterial culture was treated with colistin (1 mg/L), aztreonam (128 mg/L), and the combination of colistin and aztreonam (1 mg/L and 128 mg/L, respectively) for 1, 4 and 24 h; concentrations of colistin and aztreonam were chosen based on their MICs, pharmacokinetics in patients, and *in vitro* static time-kill results to ensure sufficient bacterial cells for the metabolomics study. The untreated bacterial culture was served as the control and five biological replicates were prepared independently from different colonies of AB090342 on different days.

### Preparation of Metabolite Samples

Cellular metabolites of AB090342 were extracted based on a previously reported method (Han et al., 2018). In brief, both treated and untreated bacterial culture (20 mL) were collected at 0, 1, 4, and 24 h and immediately quenched in a dry ice-ethanol bath for 30 s to stop metabolic processes. The culture was then normalized according to OD_600_ at 0.50 ± 0.02, 15 mL of which was centrifuged at 3,220 ×*g* and 4°C for 10 min to obtain bacterial cell pellets. After washed twice with 2 mL 0.9% sodium chloride, cell pellets were resuspended in 0.5 mL extraction solvent (CHCl_3_/MeOH/H_2_O, 1:3:1, v/v) containing 1 μM generic internal standards (CHAPS, CAPS, PIPES, and TRIS), which was followed by three freeze-thaw cycles in liquid nitrogen to lyse cells and release cellular metabolites. The supernatants (0.3 mL) containing extracted metabolites were collected after centrifugation at 3,220 ×*g* and 4°C for 10 min, then further centrifuged at 14,000 ×*g* for 10 min to achieve particle-free samples (0.2 mL) for LC-MS analysis.

### LC-MS Analysis of Metabolites

Based on a published method (Han et al., 2018), metabolite samples were analyzed on a Q-Exactive Orbitrap mass spectrometer (Thermo Fisher) coupled to a Dionex U3000 high-performance liquid chromatography (HPLC) with a ZIC-pHILIC column (5 μm, polymeric, 150 × 4.6 mm; SeQuant, Merck). The MS system was operated in both positive and negative electro-spray ionisation (ESI) mode with a resolution at 35,000 and a detection range of 85 to 1,275 *m/z*. The samples were maintained at 4°C and 10 μL of which were eluted by a multi-step gradient system which started from 20% mobile phase A (20 mM ammonium carbonate) to 80% mobile phase B (acetonitrile) to 50% A and 50% B over 15 min by a linear gradient at 0.3 mL/min. This was followed by another gradient to 5% B at 18 min and continued for 3 min before a re-equilibration with 20% A and 80% B over the next 8 min. All metabolomics samples containing internal standards were analyzed within the same LC-MS batch to minimize any potential variations. A pooled biological quality control (PBQC) sample containing an aliquot of 10 μL of each sample was analyzed periodically through the batch to monitor the chromatographic peaks, signal reproducibility and analyte stability. Eight mixtures consisting of more than 300 authentic standards were also analyzed within the batch for assisting the identification of metabolites.

### Data Processing, Statistics and Pathway Analysis

Metabolomics raw data were initially converted to mzXML files and split into both positive and negative polarity and followed by feature detection with XCMS; data from all samples were then combined and annotated using mzMatch (Smith et al., 2006; Kessner et al., 2008; Scheltema et al., 2011). The mzMatch data were filtered, identified, quantified and visualized in IDEOM using default values^1^ (Creek et al., 2012). The dataset of relative intensity (peak height) was normalized according to the median height of all putatively identified peaks, and log transformed in MetaboAnalyst 3.0 before further analysis (Xia et al., 2015). Multivariate statistics using unsupervised principle component analysis (PCA) was applied to visualize the global metabolic profiles of the samples with antibiotic treatments at each time point. Univariate statistical analysis using one-way analysis of variance (ANOVA) for multiple comparison and *post hoc* analysis using Tukey’s honestly significant difference (HSD) were conducted for the identification of significant metabolic perturbations (FDR < 0.05, *p* < 0.05, log_2_(fold change) ≥ 1 or ≤-1) between treated and untreated groups at each time point. Metabolites with an IDEOM confidence score of 6 or greater (identified by the accurate mass and standard, or predicted, retention time, corresponding to MSI level 1 or 2 based on Metabolomics Standards Initiative Guidelines), and a ≥ 2-fold change were further analyzed and subjected to pathway analysis using Kyoto Encyclopedia of Genes and Genomes (KEGG) pathway (Kanehisa and Goto, 2000), Biocyc (Karp et al., 2005) and iPath 3 (Letunic et al., 2008).

## Results

### Genomic Analysis of the Clinical Isolate *A. baumannii* AB090342

The genome sequencing produced 3,246,666 pairs of 300-bp reads for *A. baumannii* AB090342. Assembly of the genome resulted in 60 contigs larger than 500 bp, representing a 3.91 Mb draft genome. Totally, 3,683 putative coding sequences were predicted for AB090342. Our data showed that AB090342 shared 98.0% sequence similarity to colistin-susceptible *A. baumannii* AB307-0294 (Genbank Accession No: NC_011595). The genome annotation revealed that AB090342 contains multiple antibiotic resistance genes encoding OXA-51, OXA-23, metallo-beta-lactamase; aminoglycoside phosphotransferase; DNA gyrase and DNA topoisomerase IV; and TetR family transcriptional regulator responsible for tetracycline resistance. The MIC results showed consistent results with imipenem and meropenem ≥ 16 mg/L, amikacin > 128 mg/L and ciprofloxacin > 32 mg/L. Furthermore, a comparison of AB090342 and AB307-0294 revealed 1,441 variations of non-synonymous single nucleotide polymorphisms (SNPs), among which, a missense mutation of A138V and A444T in *pmrB* gene was identified. Nevertheless, the MIC results showed that AB090342 was susceptible to colistin with an MIC of 0.5 mg/L.

### Global Metabolic Variations in Response to Colistin and Aztreonam Alone and Their Combination

**FIGURE 1:**
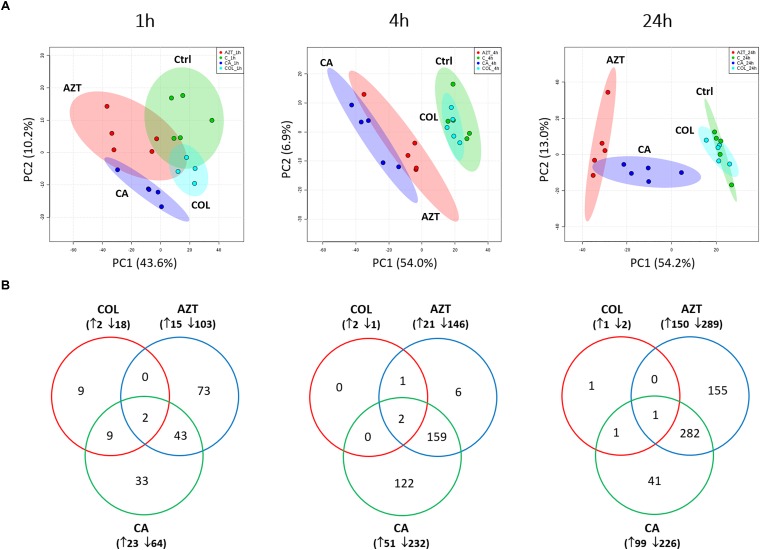
Multivariate and univariate analyses of global metabolic changes. **(A)** PCA score plots of the first two principle components for metabolite levels in *A. baumannii* AB090342 after treatments with colistin, aztreonam alone and the combination at 1, 4, and 24 h. Each dataset represents a total of 20 samples of 5 biological replicates under each condition. Green, untreated control (C); cyan, colistin alone (COL); red, aztreonam (AZT) alone; blue, combination (CA). **(B)** Venn diagrams show the number of significantly affected metabolites by each treatment at 1, 4, and 24 h. Significant metabolites were selected based on FDR < 0.05, *p* < 0.05 and FC ≥ 2 (one-way ANOVA).

**FIGURE 2:**
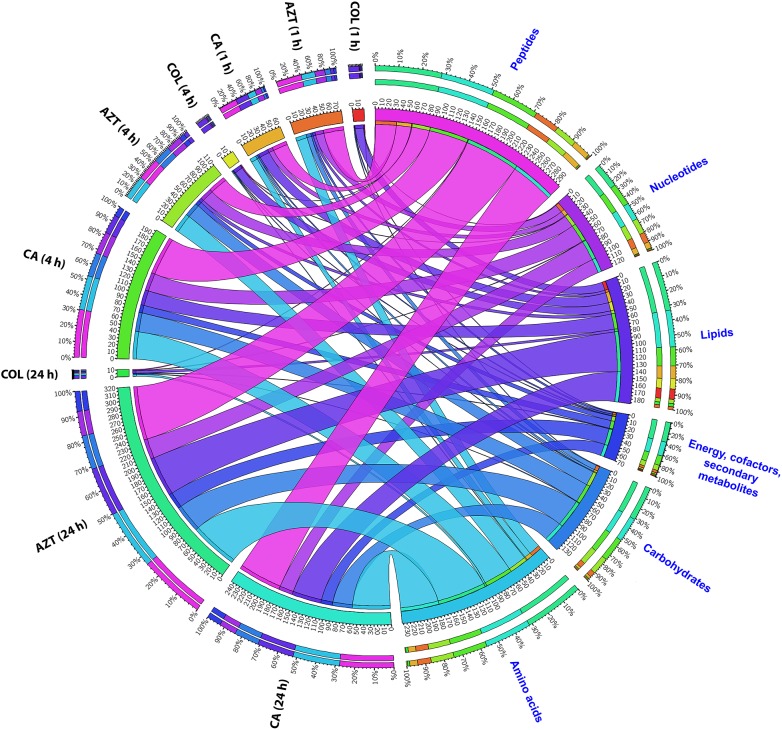
Metabolomic responses of *A. baumannii* AB090342 after the treatment with colistin (COL), aztreonam (AZT) and the combination (CA) at 1, 4, and 24 h. Bipartite graph shows the correlations of the total number and percentage of significantly affected metabolites (FDR < 0.05, *p* < 0.05 and FC > 2) in different major classes and all conditions (treatments and time points).

A total number of 1,060 putatively identified metabolites were obtained, involving in a wide range of pathways, including metabolism of amino acids, carbohydrates, lipids, nucleotides (Supplementary Dataset S1). The median relative standard deviation (RSD) value of the pooled quality control samples was 14.2%, showing minimal technical variation which was well within the acceptable limits for metabolomics (Supplementary Figure S1) (Kirwan et al., 2014). The median RSD value for each sample group (15–35%) indicated the dynamics of bacterial metabolism during *in vitro* culture (Supplementary Figure S1). As shown in the principle component analysis (PCA) score plots, the colistin and aztreonam combination induced significant metabolic changes as early as 1 h (PC1 = 43.6%), and lasted at least till 24 h (PC1 = 54.2%; Figure 1A). However, the metabolic changes induced by colistin monotherapy were only observed at 1 h. In contrast, significant metabolic perturbations caused by aztreonam monotherapy were detected at 4 and 24 h, which was even more dramatic according to the PCA analysis than the combination treatment at 24 h (Figure 1A). With regards to the number of significantly changed metabolites [FDR < 0.05, *p* < 0.05, and fold change (FC) ≥ 2, one-way analysis of variance (ANOVA)], the combination therapy resulted in 8.2% (87), 26.7% (283) and 30.7% (325) metabolic changes at 1, 4, and 24 h, respectively. Similarly, aztreonam monotherapy induced 11.1% (118), 15.8% (167) and 41.4% (439) metabolic variations across all three time points, respectively (Figures 1B, 2 and Supplementary Figure S2). However, only 1.9% (20) metabolic changes were induced by colistin alone at 1 h, and metabolism was not significantly altered at 4 or 24 h (Figures 1B, 2 and Supplementary Figure S2). In general, a large number of metabolites were shared between aztreonam monotherapy and the combination at 4 and 24 h, indicating that the synergistic killing of colistin-aztreonam combination was largely driven by aztreonam at both time points (Figure 1B).

**FIGURE 3:**
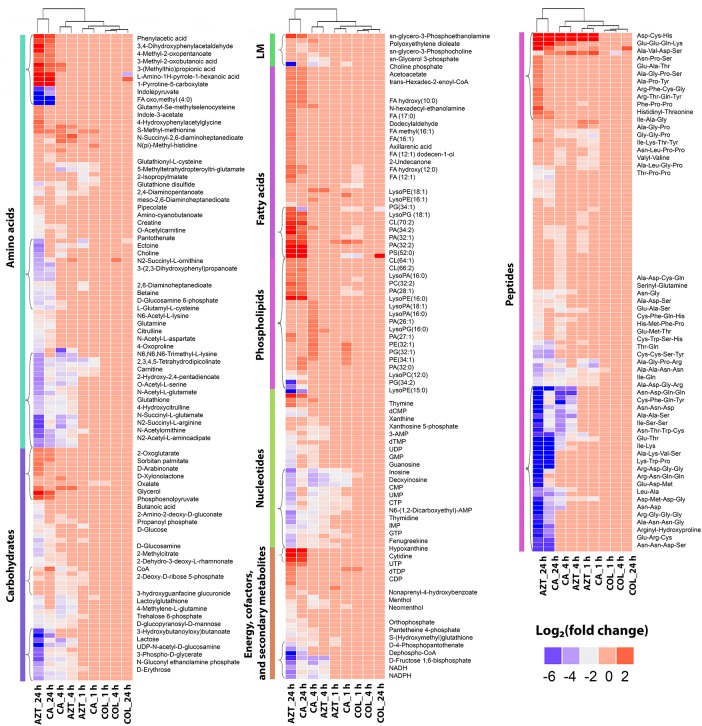
Clustered heatmap profiles of the relative abundance for significantly affected metabolites in *A. baumannii* AB090342. Metabolites are grouped into different classes: amino acids, carbohydrates, lipids (lipid metabolism [LM], fatty acids and phospholipids), nucleotides, energy, secondary metabolites and peptides. The colors indicate the relative abundance of significantly affected metabolites by all three treatments (colistin alone, aztreonam alone and the combination) at 1, 4, and 24 h compared to the untreated control samples. Metabolite names were only labeled for top-significant metabolites as shown in single brackets. Blue and gray, significantly decrease (log_2_ FC ≤ –1); red, significantly increase (log_2_ FC ≥ 1); and pink, not significant.

The metabolite enrichment analysis revealed that multiple key biochemical pathways, including nucleotide, amino acid and lipid metabolism and pentose phosphate pathway (PPP) were significantly affected in AB090342 following the treatments with colistin and aztreonam alone and the combination over 24 h (Supplementary Figure S3). In detail, both aztreonam alone and the combination significantly decreased metabolic levels in amino acid, peptide, nucleotide, pentose phosphate and amino sugar metabolism at all three time points (Figures 2, 3, Supplementary Figure S2 and Supplementary Dataset S2). On the contrary, lipid metabolism, in particular, fatty acids and glycerophospholipids (GPLs) were significantly enriched by aztreonam alone and the combination at 24 h (Figure 3 and Supplementary Dataset S2); whereas, colistin alone and the combination caused a dramatic decrease in lipid levels at 1 h (Figures 3 and Supplementary Figure S2). Furthermore, despite the significant changes caused by both aztreonam and the combination, it is notable that the combination exclusively induced significant accumulations of fatty acids and GPLs at 1 and 4 h.

### Amino Acid and Short-Chain Peptide Metabolism

**FIGURE 4:**
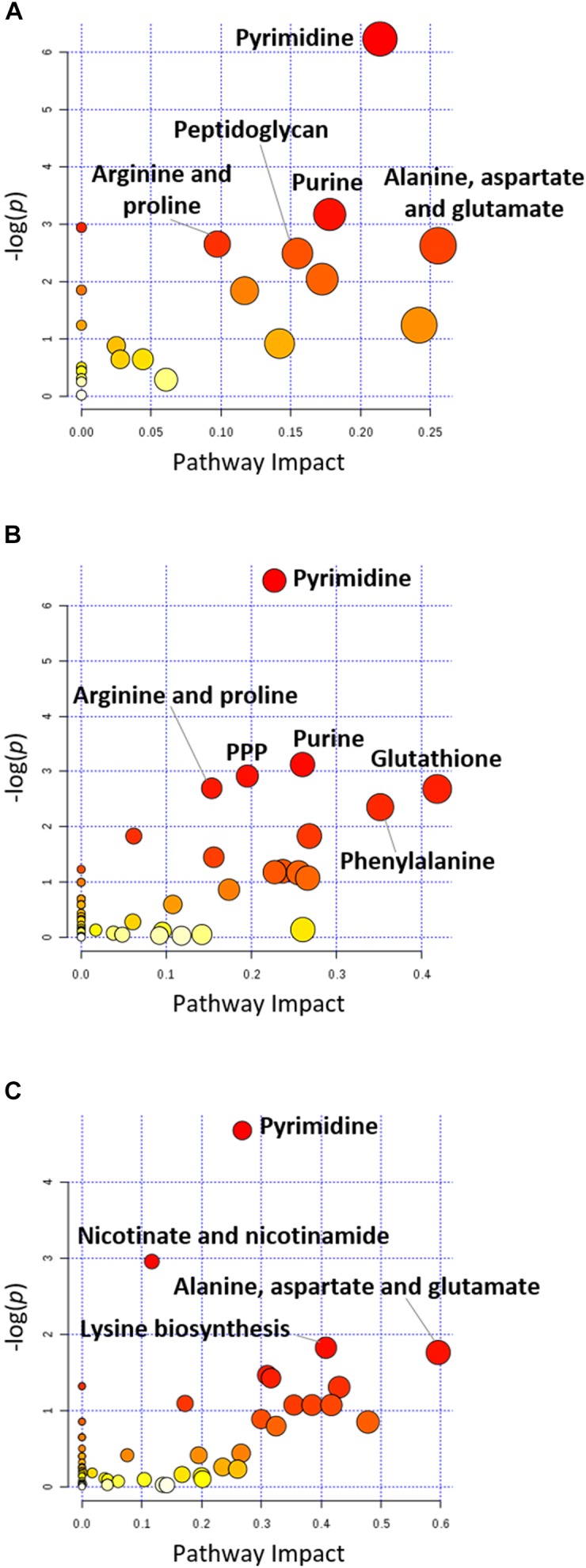
Pathway analysis of significantly affected metabolites in *A. baumannii* AB090342 following the treatments with colistin, aztreonam alone and the combination at **(A)** 1 h, **(B)** 4 h, and **(C)** 24 h (FDR < 0.05, *p* < 0.05 and FC ≥ 2, one-way ANOVA). The pathway enrichment analysis was based on KEGG Pathway (http://www.genome.jp/kegg/pathway.html) with reference to *Escherichia coli* K-12. The figure was generated by MetaboAnalyst 4.0 (https://www.metaboanalyst.ca/).

Treatment with aztreonam alone and the combination showed considerable changes in the levels of amino acids and peptides, especially at 4 and 24 h (Figures 2, 3 and Supplementary Dataset S2). In particular, the pathways related to arginine and proline (e.g., *N*-acetyl-L-glutamate, L-citrulline, 4-oxoproline and *N_2_*-succinyl-L-arginine), alanine, aspartate and glutamate (e.g., *N*-acetyl-L-aspartate and *O*-acetylcarnitine), glutathione (e.g., glutathione and glutathione disulfide), and lysine (e.g., *N_2_*-acetyl-L-aminoadipate and *N*-succinyl-2,6-diaminoheptanedioate) metabolism were all significantly decreased due to aztreonam monotherapy and the combination treatment at 4 and 24 h (log_2_FC ≤-1) (Figures 3, 4 and Supplementary Dataset S2). In contrast, the levels of phenylacetic acid, 4-methyl-2-oxopentanoate, and 3-(methylthio)propionic acid were dramatically increased in response to both aztreonam alone and the combination treatments (log_2_FC > 1) at 24 h. Interestingly, the abundance of several metabolites associated with tryptophan metabolism (e.g., indole-3-acetate, indolepyruvate and isophenoxazine) was elevated following treatment with aztreonam alone or the combination over 24 h. Consistently, a substantial perturbation in the levels of short-chain peptides was also observed due to aztreonam alone and the combination treatments. Apparently, aztreonam monotherapy induced more metabolic changes in amino acid and peptide levels at 24 h compared to the combination treatment (Figures 2, 3).

### Pentose Phosphate Pathway and Amino Sugar Metabolism

**FIGURE 5:**
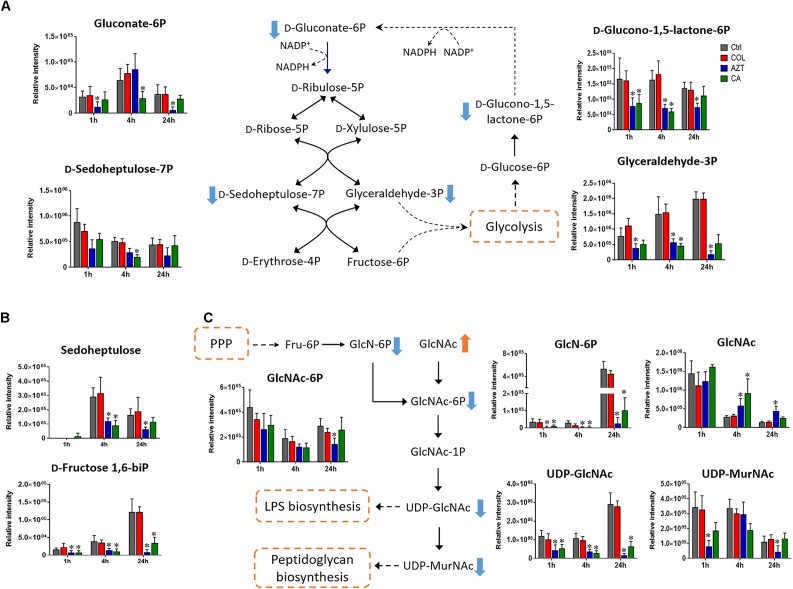
Metabolic changes in **(A)** pentose phosphate pathway (PPP), **(B)** metabolites associated with PPP and carbon fixation, and **(C)** amino sugar metabolism in *A. baumannii* AB090342 after the treatment with colistin (COL, red), aztreonam (AZT, blue) and the combination (CA, green) compared to untreated control (Ctrl, gray) at 1, 4, and 24 h. The pathway flow charts were adapted from KEGG Pathway (http://www.genome.jp/kegg/pathway.html) with reference to *Escherichia coli* K-12. Orange and blue arrows indicate that the metabolites were significantly increased and decreased, respectively. ^∗^FDR < 0.05, *p* < 0.05 and log_2_ FC ≥ 1 or ≤ –1 (one-way ANOVA).

Central carbon metabolism was significantly decreased by aztreonam alone and the combination, with major changes observed for the metabolites associated with bacterial anabolic metabolism of the PPP at 1, 4, and 24 h. A number of key metabolites (e.g., gluconate 6-phosphate, D-sedoheptulose 7-phosphate, glyceraldehyde 3-phosphate and D-glucono-1,5-lactone 6-phosphate) were significantly decreased (log_2_FC ≤-1) in their abundance in response to aztreonam alone and the combination (Figure 5A). Moreover, the levels of sedoheptulose (log_2_FC = -1.3 to -2.0) and D-fructose 1,6-biphosphate (log_2_FC = -1.4 to -3.9) related to carbon fixation and PPP metabolism were also significantly decreased at 4 and 24 h (Figure 5B). In addition, a significant perturbation in the amino sugar metabolism was also observed after the treatments of aztreonam alone and the combination at all three time points (Figure 5C). In detail, the decreased relative abundance of three metabolites (i.e., D-glucosamine, D-glucosamine 6-phosphate and *N*-acetyl-D-glucosamine 6-phosphate) was observed (log_2_FC < -1), which resulted in the decreased level of uridine diphosphate-*N*-acetylglucosamine (UDP-GlcNAc), an important precursor for the synthesis of lipopolysaccharide and peptidoglycan. The decreased metabolite level in this pathway resulted in the accumulation of *N*-acetyl-D-glucosamine (GlcNAc) at 4 and 24 h under aztreonam alone (log_2_FC = 1.1 and 1.6, respectively) and the combination (log_2_FC = 1.7 and 0.8, respectively). Moreover, at 1 h aztreonam monotherapy significantly decreased the level of UDP-*N*-acetylmuramate (UDP-MurNAc, log_2_FC = -2.1), another key metabolite associated with peptidoglycan synthesis (Figures 4, 5C).

### Perturbations in Fatty Acid and Phospholipid Levels

**FIGURE 6:**
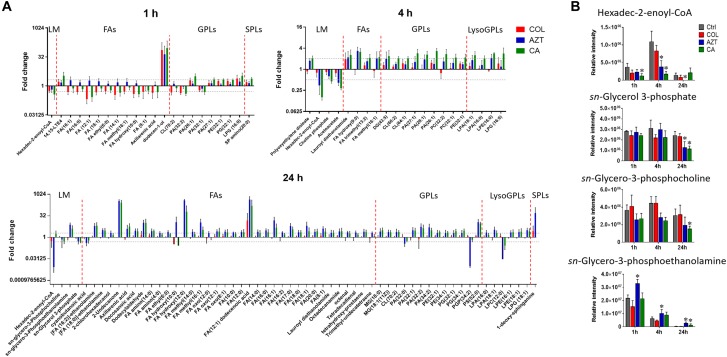
Perturbations of lipids and the related metabolites in *A. baumannii* AB090342. **(A)** Significantly perturbed major lipid classes following the treatment with colistin (COL, red), aztreonam (AZT, blue), and the combination (CA, green) at 1, 4, and 24 h. Lipid names were putatively identified based on the accurate mass. LM: lipid metabolism; FAs: fatty acids; GPLs: glycerophospholipids; SPLs: sphingolipids. **(B)** Significantly perturbed metabolites related to fatty acid and phospholipid synthesis and degradation after treated by colistin, aztreonam and the combination across all three time points. ^∗^FDR < 0.05, *p* < 0.05 and log_2_ FC ≥ 1 or ≤ –1 (one-way ANOVA).

Treatments with colistin alone and the combination with aztreonam significantly disrupted bacterial lipids at 1 h; in particular, the medium-chain fatty acids [FA (12:1) and FA hydroxyl (10:0)] and glycerophosphates [PA (32:0) and PA (32:2)] were depleted significantly (log_2_FC < -1) (Figure 6A). Interestingly, the combination treatment significantly enriched a number of GPLs, in particular, phosphatidylethanolamine [PE (32:1)], phophatidylglycerol [PG (32:1)] and cardiolipin [CL (64:1 and 66:2)] over 24 h (log_2_FC > 1). Obviously, aztreonam monotherapy elevated fatty acid levels more dramatically compared to the combination treatment, whereas, long-chain GPLs, including PA (26:1 and 32:1), PE (32:0, 32:1, and 34:1) and PG (32:1 and 34:1) were more significantly enriched by the combination at 24 h (Figure 6A and Supplementary Dataset S2). In addition, our results also showed that significant perturbations of metabolites related to fatty acid elongation and degradation (hexadec-2-enoyl-CoA) and GPLs biosynthesis and degradation (*sn*-glycero-3-phosphate, *sn*-glycero-3-phosphocholine and *sn*-glycero-3-phosphoethanolamine) were induced by either aztreonam alone or the combination over 24 h (Figure 6B).

### Purine and Pyrimidine Metabolism

**FIGURE 7:**
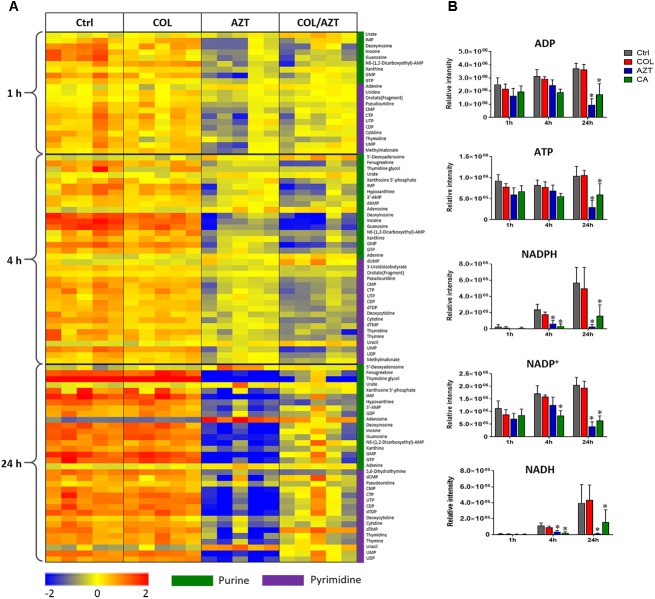
Perturbations of nucleotide metabolism. **(A)** Heatmap profiles of the relative abundance of nucleotide metabolites after treated with colistin (COL), aztreonam (AZT) and the combination (COL/AZT) at 1, 4, and 24 h. **(B)** Significantly altered metabolites associated with nucleotide metabolism due to aztreonam alone and the combination of colistin and aztreonam at 1, 4, and 24 h in *A. baumannii* AB090342. ^∗^log_2_ FC ≥ 1 or ≤ –1, *p* < 0.05, FDR < 0.05 (one-way ANOVA). Metabolomics data were collected from five biological replicates per condition.

Metabolite levels related to purine and pyrimidine metabolism were significantly decreased by either aztreonam alone or the combination across all three time points (Figures 4, 7A). However, adenosine and uracil were significantly enriched at 24 h by aztreonam monotherapy (log_2_FC = 3.2 and 1.6, respectively) and the combination treatment (log_2_FC = 2.1 and 0.8, respectively). Notably, compared to aztreonam alone, the combination induced more dramatic changes in nucleotide metabolism at 4 h, but less at 24 h (Figure 7A). Specifically, two important energy sources, adenosine diphosphate (ADP) and adenosine triphosphate (ATP) (log_2_FC = -1.1 to -2.0) at 24 h, and three nucleotide-derived metabolites related to redox status, nicotinamide adenine dinucleotide phosphate (NADP^+^), NADPH and NADH at 4 and 24 h (log_2_FC = -1.0 to -4.9) were all dramatically depleted after the treatments of aztreonam alone and the combination at 24 h (Figure 7B).

## Discussion

Polymyxin combination therapies with other antibiotics have been demonstrated synergistic against MDR *A. baumannii* by increasing bacterial kill and reducing emergence of resistance (Cai et al., 2012; Vidaillac et al., 2012; Bae et al., 2016). In the present study, we investigated the metabolic responses of an *A. baumannii* clinical isolate under the treatments with colistin and aztreonam alone and in combination over 24 h. Significantly, our metabolomics results revealed that at the tested concentrations: (1) colistin-facilitated while aztreonam-dominated metabolic perturbations; (2) time-dependent pathway alterations; (3) changes in PPP and the downstream lipid, amino acid and nucleotide metabolism; and (4) synergistic inhibition of cell envelope synthesis and alterations in the membrane phospholipid composition by the combination.

Our genome sequencing data for AB090342 revealed a mutation of two bases, an in-frame mutation of A138V and A444T, in *pmrB* which has not been characterized before. It is known that polymyxin resistance in *A. baumannii* can be associated with mutations in *pmrB* which upregulate the phosphoethanolamine transferase, EptA, and subsequently result in lipid A modification (Moffatt et al., 2010; Arroyo et al., 2011). Although both mutations in *pmrB* did not cause lipid A modification or polymyxin resistance in AB090342 (colistin MIC = 0.5 mg/L), the constant exposure to colistin in the clinic may have resulted in resistance in clinical isolates. Therefore, colistin combination therapies with other antibiotics are strongly recommended to increase the killing effect and minimize the emergence of resistance.

Our metabolomics results showed that the synergistic action of colistin-aztreonam combination resulted in metabolic alterations in lipid, carbohydrate, nucleotide, amino acid and peptide metabolism. The initial metabolic perturbations following colistin monotherapy and the combination at 1 h mainly involved lipid metabolism (13 out of 20 significant metabolites), in particular the significantly decreased levels of fatty acids and increased phospholipids (Figures 3, 6A). Colistin displays its antimicrobial activity through the initial target LPS on the Gram-negative OM, which results in the increased OM permeability and phospholipid exchange (Velkov et al., 2013; Rabanal and Cajal, 2017). Notably, the significantly perturbed lipid levels by the combination at 1 h were consistent with those observed for colistin alone, suggesting the membrane-targeted killing mechanism. Our results are consistent with previous transcriptomics and metabolomics findings in *A. baumannii* that colistin significantly disturbed OM asymmetry and up-regulated expression of the Mla system which is responsible for phospholipid transfer (Henry et al., 2015; Maifiah et al., 2017). On the contrary, aztreonam alone did not produce any significant alterations in lipid levels at 1 h, but considerably depleted metabolites in amino acid, central carbon and nucleotide metabolism. The mode of action of aztreonam is through interaction with penicillin binding protein 3 (PBP3) which leads to the inhibition of bacterial cell wall synthesis (Ramsey and MacGowan, 2016). Consistent with this primary mechanism, aztreonam monotherapy for 1 h resulted in dramatic decrease in the intracellular level of UDP-MurNAc which is an important precursor for peptidoglycan synthesis (Figures 3, 4, 5C).

The metabolic responses of *A. baumannii* AB090342 to colistin and aztreonam monotherapy and the combination indicated time-dependent metabolic alterations over 24 h (Figures 2, 3, Supplementary Figure S2 and Supplementary Dataset S2). Aztreonam alone and the combination at 4 and 24 h significantly decreased metabolic levels in amino acid, carbohydrate and nucleotide metabolism, but increased lipid levels. However, colistin alone failed to produce any significant metabolic alterations at 4 or 24 h. The largely shared metabolic changes between aztreonam monotherapy and the combination demonstrated that the synergistic killing by colistin-aztreonam combination was mainly driven by aztreonam at 4 and 24 h. The treatments of aztreonam alone and the combination significantly perturbed metabolite levels in the synthesis of amino sugars at 4 and 24 h, in particular, UDP-GlcNAc and UDP-MurNAc which are important precursors for LPS and cell wall biosynthesis, consistent with the antibacterial activity of aztreonam (targeting cell wall) and colistin (targeting cell envelope) (Cho et al., 2014; Ramsey and MacGowan, 2016; Maifiah et al., 2017; Han et al., 2018). In addition to disturbing cell wall biosynthesis, aztreonam also severely depleted the levels of metabolites related to amino acid and nucleotide metabolism which is consistent with the substantially decreased peptide levels (Figures 3, 7A). Moreover, the decreased metabolite levels in PPP coupled to the lower levels of NAD metabolites suggested an imbalanced redox state within bacterial cells treated by aztreonam alone and the combination (Ying, 2008). In contrast, the fatty acid and phospholipid levels were significantly elevated by aztreonam monotherapy and the combination at 4 and 24 h, which was possibly due to the reduced utilization as an energy source, decreased cell turn over and/or membrane remodeling (Figures 3, 6; Lobritz et al., 2015).

Notably, despite the largely shared metabolic perturbations with colistin monotherapy at 1 h and aztreonam monotherapy at 4 and 24 h, the combination displayed the greatest metabolic changes at 4 h and a number of unique metabolic alterations at each time point (Figures 1, 3 and Supplementary Figure S2). In particular, the combination displayed synergy as early as 1 h and lasted for at least 24 h, which was shown by the significant changes in the membrane phospholipid composition that PA, PE, PG and cardiolipin were more dramatically enriched by the combination compared to either monotherapy over 24 h (Figures 3, 6). It is also evident that the combination synergistically inhibited the LPS and cell wall synthesis (Figure 5C). Interestingly, lipid A modification pathways were not affected at any time point under neither of the conditions investigated here, which was also reported for the combination of polymyxin B and doripenem against *A. baumannii* (Maifiah et al., 2017). Collectively, these results show that the combination synergy between polymyxins and *β*-lactams is mainly due to the inhibition of cell envelope, but not the prevention of lipid A modification mediated polymyxin resistance.

Taken together, our metabolomic results demonstrated, for the first time, that the time-dependent synergistic killing against *A. baumannii* by colistin-aztreonam combination was initially driven by colistin and subsequently by aztreonam through inhibiting multiple key biochemical pathways. This study provides important mechanistic information for optimizing colistin-aztreonam combination therapy in patients using pharmacokinetics/pharmacodynamics.

## Author Contributions

JZ and JL conceived the project. M-LH and XL performed the experiments, and M-LH, XL, TV, Y-WL, ML, YZ, and DC analyzed the results. All authors involved in the design of the experiments and reviewed the manuscript.

## Conflict of Interest Statement

The authors declare that the research was conducted in the absence of any commercial or financial relationships that could be construed as a potential conflict of interest.

## Footnotes

1 http://mzmatch.sourceforge.net/ideom.php
