# Supplementary material for: Metabolic Analyses Revealed Time-Dependent Synergistic Killing by Colistin and Aztreonam Combination Against Multidrug-Resistant Acinetobacter baumannii
Source: Front Microbiol. 2018 Nov 16;9:2776. doi: 10.3389/fmicb.2018.02776 (PMC6250834; doi:10.3389/fmicb.2018.02776)
Supplement: Supplementary file 1 [file Data_Sheet_1.docx]

**Supporting Information**

**Metabolic analyses revealed time-dependent synergistic killing by colistin and aztreonam combination against multidrug-resistant *Acinetobacter baumannii***

Mei-Ling Han^1,2,#^, Xiaofen Liu^2,#^, Tony Velkov^3^, Yu-Wei Lin^1^, Yan Zhu^1^, Mengyao Li^1^, Heidi H. Yu^1^, Zhihui Zhou^4^, Darren J. Creek^5^, Jing Zhang^2,*^, Jian Li^1,2^

^1^Biomedicine Discovery Institute, Infection and Immunity Program, Department of Microbiology, Monash University, Clayton, Victoria 3800, Australia.

^2^Institute of Antibiotics, Huashan Hospital, Fudan University, Shanghai 200040, China.

^3^Department of Pharmacology & Therapeutics, School of Biomedical Sciences, Faculty of Medicine, Dentistry and Health Sciences, The University of Melbourne, Parkville, Victoria 3010, Australia.

^4^Department of Infectious Diseases, Sir Run Run Shaw Hospital, Zhejiang University School of Medicine, Hangzhou, China.

^5^Drug Delivery, Disposition and Dynamics, Monash Institute of Pharmaceutical Sciences, Monash University, 381 Royal Parade, Parkville, Victoria 3052, Australia.

^#^Contributed equally to this work.

*Joint senior authors. Address correspondence to: 12 Wulumuqi Zhong Rd, Shanghai, 200040, China; Email: [zhangj_fudan@aliyun.com](mailto:zhangj_fudan@aliyun.com); Phone: 86-21-52888190.

**Running title:** Colistin and aztreonam against *Acinetobacter baumannii*.


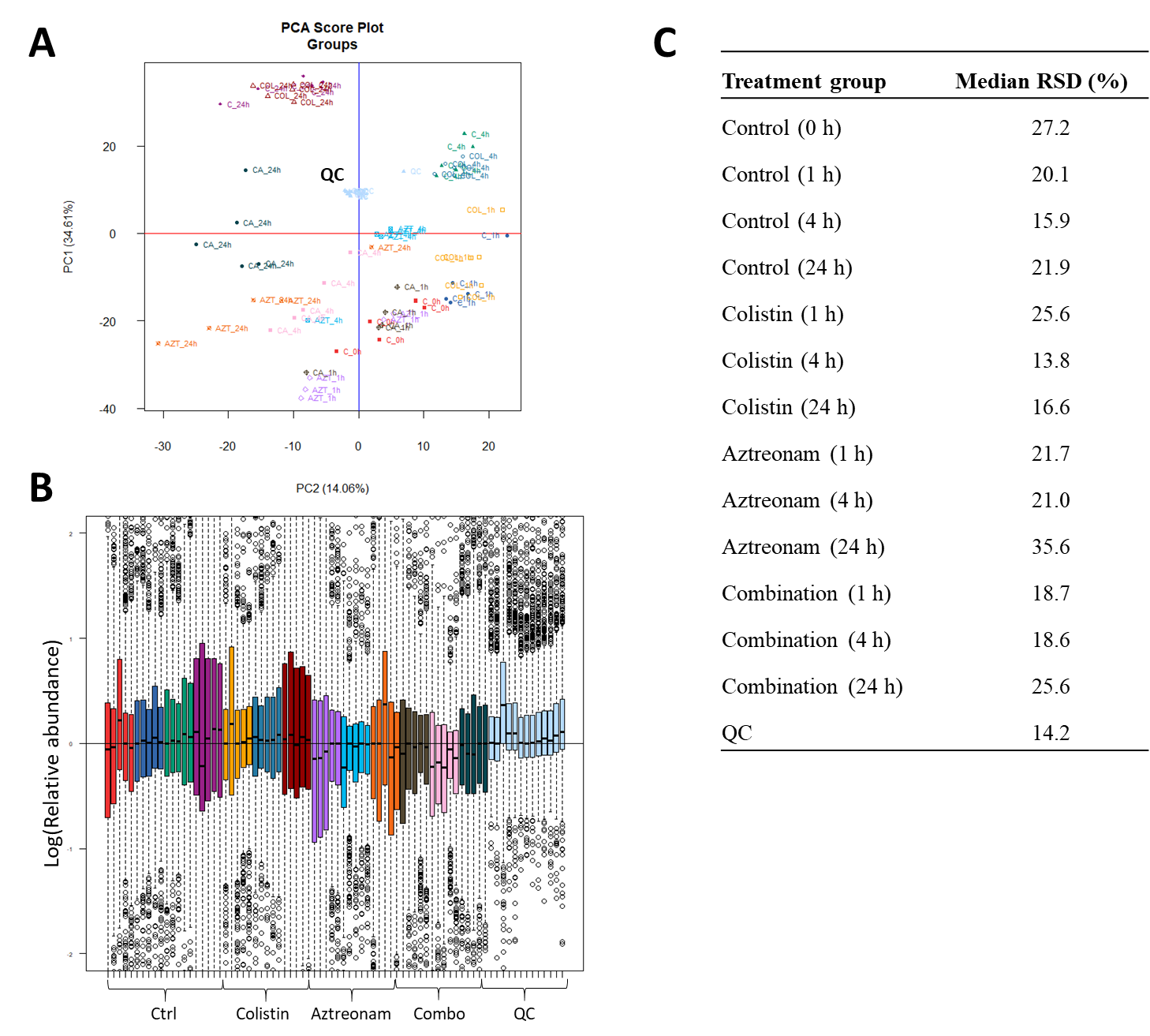


**Figure S1.** **(A)** PCA score plot of all metabolites from the intracellular metabolite and QC samples of *A. baumannii* AB090342; **(B)** Log-transformed relative abundance of each sample; and **(C)** Data precision of individual samples represented as the median relative standard deviation (RSD) for all metabolites based on all replicates of each group. Four quality controls (QCs) were analyzed within the same LC-MS batch with treated and untreated samples. Each dataset represents five biological replicates.


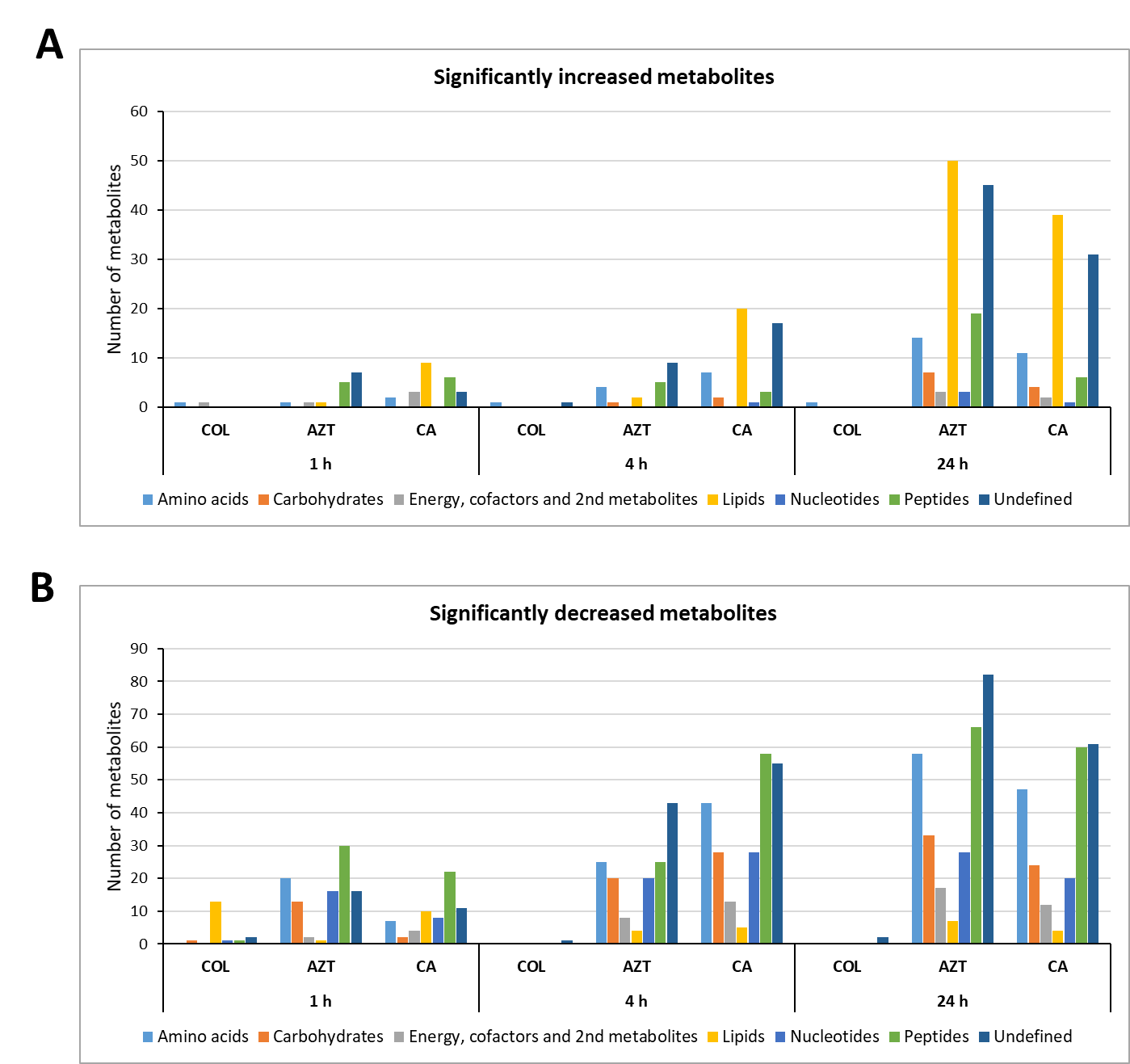


**Figure S2.** Numbers of significantly increased and decreased metabolites in major metabolic pathways in *A. baumannii* AB090342 due to treatments with colistin (COL), aztreonam (AZT) and the combination (CA) at 1, 4 and 24 h (FDR < 0.05, *p* < 0.05, and fold change (FC) ≥ 2, one-way ANOVA).


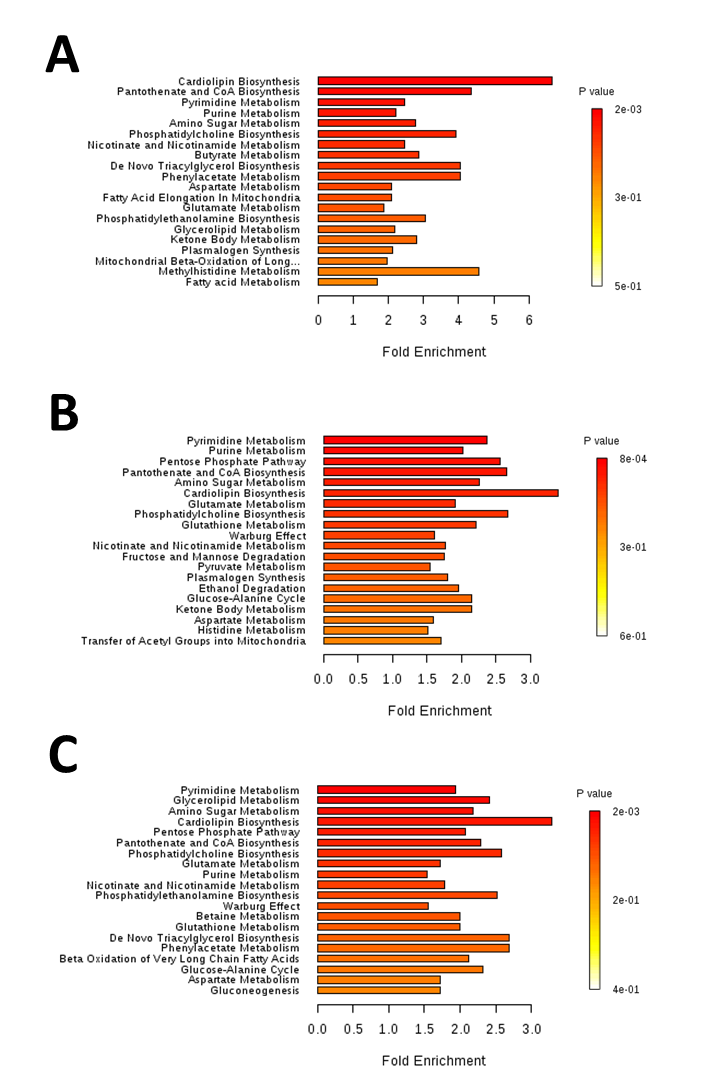


**Figure S3.** Top 20 perturbed pathways identified by enrichment analysis in *A. baumannii* AB090342 due to treatments with colistin, aztreonam and the combination at **(A)** 1 h, **(B)** 4 h and **(C)** 24 h (FDR < 0.05, *p* < 0.05, and FC ≥ 2, one-way ANOVA).
